# Suicidal Ideation and Traumatic Exposure Should Not Be Neglected in Epileptic Patients: A Multidimensional Comparison of the Psychiatric Profile of Patients Suffering From Epilepsy and Patients Suffering From Psychogenic Nonepileptic Seizures

**DOI:** 10.3389/fpsyt.2019.00303

**Published:** 2019-05-03

**Authors:** Abel Guillen, Jonathan Curot, Philippe Jean Birmes, Marie Denuelle, Valérie Garès, Simon Taib, Luc Valton, Antoine Yrondi

**Affiliations:** ^1^Explorations Neurophysiologiques (Neurophysiological Investigations), Hôpital Pierre Paul Riquet, CHU Purpan (Toulouse University Hospital), Toulouse, France; ^2^Centre de Recherche Cerveau et Cognition (Brain and Cognition Research Centre), University of Toulouse, Toulouse, France; ^3^Centre National de la Recherche Scientifique CerCo (CerCo National Scientific Research Centre), Toulouse, France; ^4^Toulouse NeuroImaging Centre, University of Toulouse, Inserm, UPS, Toulouse, France; ^5^University of Rennes, INSA, CNRS, IRMAR-UMR 6625, Rennes, France; ^6^Service de Psychiatrie et Psychologie Médicale (Department of Psychiatry and Medical Psychology), CHU de Toulouse (Toulouse University Hospital), Toulouse, France

**Keywords:** PNES, epilepsy, suicidal behavior, suicide, trauma, PTSD

## Abstract

**Introduction:** Patients with psychogenic nonepileptic seizures (PNESs) have often been exposed to traumatic events, which is a risk factor for suicidal behavior. This would suggest that the severity of suicidal ideation is greater in PNES than in patients suffering only from epileptic seizures (ESs). However, these psychiatric symptoms may be underestimated in the ES population. The specific features or similarities between the psychiatric clinical profiles of these two groups should be elaborated to improve therapeutic management. Our study is the first to compare suicidal ideation, suicide risk, posttraumatic stress disorder (PTSD), and depression disorder simultaneously in both groups, in a tertiary care epilepsy center.

**Material and methods:** We prospectively enrolled patients hospitalized for video-electroencephalography (EEG) monitoring to assess repeated seizures before an ES or a PNES diagnosis was made. During the psychiatric consultation that accompanied the video EEG, we rated the severity of suicidal ideation and depressive symptoms, suicidal risk, traumatic exposure history, and PTSD symptoms.

**Results:** Eighteen subjects were enrolled and diagnosed with PNES, and 42, with ES. The PNES group reported more exposures to traumatic events and more intense PTSD symptoms (median: 17 vs. 27; *p* = 0.001). The severity of suicidal ideation did not differ significantly between the two groups.

**Conclusion:** It is the severity of PTSD symptoms in PNES patients that differentiates them from ES patients, although exposure to traumatic events is also frequent in ES patients. We demonstrated that suicidal ideation and suicide risk are equally high in the ES and PNES groups. Therefore, both groups require extreme vigilance in terms of suicidal risk.

## Introduction

A variety of interactions between epilepsy and psychiatric disorders have been described. One major interaction concerns the occurrence of psychogenic nonepileptic seizures (PNESs). As many as 30% of the cases in epilepsy centers for drug-resistant epilepsy appear to be due to PNES (1).

PNESs are defined as paroxysmal, transient clinical episodes that can include motor, sensory, vegetative, psychological, and cognitive signs that resemble those seen in epileptic seizures (ESs). PNESs, or “conversion disorder with attacks or seizures,” are classified by the *Diagnostic and Statistical Manual of Mental Disorders, 5th Edition* (DSM-5) as *somatic symptom disorders and related disorders* (2), a category based on the presence of positive symptoms and not simply the exclusion of an “organic” diagnosis. Video electroencephalography (vEEG) allows the clinical and electrical differences between PNES and ES to be documented; with the history of patients and witnesses of the clinical events, it offers a diagnostic “gold standard” with high levels of certainty and excellent interrater reliability (IRR) (3, 4). However, PNES and ES are not mutually exclusive. Approximately 10% of patients suffering from PNES are also afflicted by epilepsy (ES/PNES cases) (5).

Only a few studies (6–11) have examined the relationship between traumatic exposure and subsequent posttraumatic stress disorder (PTSD) symptoms in patients suffering from PNES, even though studies show that up to 80% of these patients report previous traumatic events (from 44% to 100%) (12). Among these events, maltreatment (11) and child sexual abuse (13) were more common in patients suffering from PNES than in patients suffering from ES. However, despite their high rates among PNES patients, traumatic events are not mandatory. This background significantly increases their distress and handicap. In addition, Myers et al. (14) showed that patients with PNES diagnoses exhibited significantly higher rates of sexual and “other” trauma compared with those with intractable epilepsy. However, subgroup analyses revealed that a history of psychological trauma was the only variable found to discriminate between patients with PNES and those with epilepsy.

Moreover, this strong correlation between PNES and PTSD explains some common points between the two diseases such as alterations in autonomic and subjective responsivity (15, 16). In the past, these common points led to similarities between PNES and PTSD in the management of the disease: for example, prolonged exposure therapy showed efficacy in decreasing both PNES and PTSD symptoms (17).

However, the relationship between PNES and ES is certainly not the only interrelation between epilepsy and psychiatric disorders (18). One in three epileptic patients develop other psychiatric symptoms during their lifetime (19), whereas the proportion in the general population is one in five. A 3- to 5-fold increase in suicide risk is seen in ES patients compared to the general population, and a 25-fold increase in cases of temporal lobe epilepsy (20–22). Among ES patients, the presence of suicidal ideation is greatly increased in those who also have psychiatric comorbidities (OR = 21.6) (23). In one pediatric epilepsy ward, children diagnosed with PNES or ES/PNES reported significantly more suicidal ideation than did those suffering from ES alone (24). It is more equivocal in the adult population, where contradictory results are found since no difference between ES and PNES is observed (25, 26). For instance, D’Alessio et al. (25) did not observe any difference in suicide attempts between the two groups. Such a result may be explained by a lack of statistical (secondary objectives) power, whereas suicidal ideation is part of the primary study endpoint in the pediatric cohort (24).

Although these different interrelations have been reported, gaps exist in the available literature concerning the specific features and similarities between the psychiatric profiles of ES and PNES. On one hand, to our knowledge, no study that compares the extent of suicidal ideation in ES and PNES patients has been conducted to date. On the other hand, we lack valuable data concerning the predisposing factors for PNES and its comorbidities. These gaps can make it difficult to identify psychiatric symptoms such as suicidal ideation in both ES and PNES populations in routine clinical practice. Their identification is a real therapeutic challenge.

In this study, we attempted to fill these gaps and refine these psychiatric profiles by comparing the severity of suicidal ideation in a group of ES patients vs. a group of PNES patients. It has been demonstrated that exposure to traumatic events is a risk factor for suicidal behavior (18). Therefore, we hypothesized that the severity of suicidal ideation is greater in PNES patients than in ES patients. Similarly, there is a correlation between traumatic events, PTSD, and suicide risk (27). We also examined the possibility of a link between traumatic exposure, PNES, and suicide risk, which has never been demonstrated. To the best of our knowledge, our study is the first to compare suicidal ideation, suicide risk, PTSD, and depression disorders simultaneously in a group of ES patients and a group of PNES patients in a tertiary care epilepsy center in order to refine the clinical profiles of both groups of patients. Better knowledge of these clinical profiles should raise awareness among clinicians and lead to more appropriate management strategies.

## Materials and Methods

### Subjects and Protocol

This prospective study included patients consecutively investigated at the Toulouse University epilepsy unit from August 2015 to May 2016. Patients were told about the study by the neurologists (LV and MD) and gave their informed consent on admission to the tertiary epilepsy center at Toulouse University Hospital for long monitoring video-electroencephalography (LMVEEG). This study was approved by the Toulouse University Hospital Ethics and Research Committee (no. 04615). The inclusion criteria were as follows: LMVEEG (1 to 5 days) and no objection after receiving the information leaflet. The exclusion criteria were as follows: under 15 or over 65 years of age, lack of fluency in French, any neurodegenerative disorder, or anyone subject to a legal protection measure (tutelage, trusteeship, or legal protection). Before ES or PNES was diagnosed, a psychiatrist (AG) assessed the enrolled patients in order to establish the severity of suicidal ideation, the risk of suicide, the severity of symptoms of depression, the history of traumatic exposure, and the severity of PTSD symptoms.

ES or PNES or ES/PNES was diagnosed by epileptologists, based on a thorough and comprehensive analysis of information on the semiology of the “seizures”: detailed description by patients and close relatives, detailed description by an expert clinician, analysis of the video, and complete analysis of the LMVEEG. Usual neurological activating procedures (such as hyperventilation, photic stimulation, and sleep deprivation) have been systematically used in all patients during the LMVEEG. No specific suggestion technique and no induction techniques (pharmacologic or saline injections) were used.

The diagnosis is considered as documented, clinically based, probable, or possible, in accordance with the recommendations of the International League Against Epilepsy Non-epileptic Seizures Task Force (28). All patients were enrolled when hospitalized for LMVEEG. Therefore, we used the same methodology to classify PNES and ES. The diagnosis was classified as documented (only if the seizure was recorded with LMVEEG), clinically based (based on the visual analysis of seizure semiology by the epileptologist, either live or after replaying a different video recording from the LMVEEG), probable (based on the visual analysis of the seizure semiology by the other medical doctor who either reviewed the video recording or witnessed the seizure in person, with compatible interictal EEG and MRI results, without LMVEEG), or as possible (based on retrospective analysis of the description of the seizure semiology by a witness, with compatible interictal EEG and MRI results). Epileptologists were blinded to the results of all psychiatric questionnaires. They were, however, informed of the presence of psychiatric comorbidities, to adapt the treatment management. They are aware that all patients (with ES or PNES) are at risk of psychiatric comorbidities. The diagnosis of PNES or ES was based only on the analysis of the clinical semiology of the “seizure.”

### Measures

#### Suicidal Ideation

The severity of suicidal ideation was measured using Beck’s Scale for Suicide Ideation (SSI) (29). This scale comprises 19 items, each rated from 0 to 2. The total score ranges from 0 to 38. The higher the score, the more severe the suicidal ideas. Increasing scores reflect greater suicide risk, and any positive response merits investigation (30). The first five items are screening questions that provide an assessment of the patient’s desire to live or die, including the wish to end their own life. If active or passive suicidal ideation is observed, the other questions are asked to assess the duration and frequency as well as any preparation in relation to the planned suicide attempt. It measures the current severity of suicidal attitudes, behaviors, and plans with a good predictive validity for suicide (31, 32). A score of 6 or higher was used as a cutoff threshold for clinically significant suicidal ideation (33).

#### Suicide Risk

Suicide risk was assessed using module C of the Mini International Neuropsychiatric Interview (MINI 5.0.0) (34). This structured diagnostic interview explores the main axis I psychiatric disorders of *DSM-IV*. Module C measures the current suicide risk: absent, low, moderate, or high.

#### Symptoms of Depression

Severity of depression symptom was assessed using the Beck Depression Inventory–Short Form (BDI-SF) (35). This is a self-assessment scale with 13 items. Each item is rated from 0 to 3. The total score ranges from 0 to 39. The higher the score, the greater the severity of depressive symptoms. The usual severity thresholds (36) were used:

- 0 to 4: no depression
- 4 to 7: mild depression
- 8 to 15: moderate depression
- 16 to 39: severe depression

#### Traumatic Exposure

This was assessed using the Life Events Checklist from the Clinician-Administered PTSD Scale (CAPS) (37). It identifies difficult or stressful situations a person may have had to endure. For each event, the subject indicates a) that he/she has personally experienced the situation, or b) that he/she has witnessed such a situation being experienced by another person, or c) that he/she has learned that such a situation has happened to a relative or friend. The worst event was chosen as the index event for assessment of PTSD symptoms.

#### Symptoms of PTSD

To assess PTSD symptoms, the Posttraumatic Stress disorder Check-List Specific (PCL-S) was used. At the time of the study, no tool was available for assessing PTSD according to *DSM-5* criteria that had been validated in French. The PCL-S is a 17-item instrument that parallels diagnostic Criteria B (reexperiencing), C (avoidance), and D (hyperarousal) for PTSD, as delineated in the *DSM-IV* (38). The PCL-S was designed for use as a self-reporting instrument that closely assesses each of the 17 *DSM*-defined PTSD symptoms separately (39). Each item is rated on a five-point Likert scale (1 “not at all” to 5 “very often”). Total scores range from 17 to 85, with higher scores reflecting increased levels of PTSD symptoms. In our study, we considered two statuses according to total PCL-S scores:

- Scores >44 corresponded to a “probable PTSD” and reflected a condition deserving clinical attention (38, 39).
- “Partial PTSD” includes some symptoms of PTSD but not enough to classify it as a formal diagnosis of “probable PTSD.” It was defined as a report of at least one symptom in Criteria B, C, and D (40, 41).

### Statistical Analyses

Statistical analyses were conducted with SPSS 20.0 (SPSS Inc., Chicago, IL) and R version 3.5.2 (42).

Given that there were few participants (fewer than 50 per group), a Shapiro–Wilk test was used to test the null hypothesis that the samples were normally distributed. The assumption of normality was verified with respect to age and the number of significant events. However, it was rejected with respect to the scores for the three psychometric scales: SSI, BDI-SF, and PCL-S. Nonparametric tests were required to analyze these data.

For the primary objective, a Wilcoxon–Mann–Whitney test was used to compare the SSI distributions in the two groups (ES vs. PNES).

For the secondary objectives:

A Wilcoxon–Mann–Whitney test was used to compare the distributions of the scores for BDISF and for PCL-S and the number of significant events in the two groups.
A Pearson χ2 test was used to examine the association between the two groups and the results of module C of the MINI 5.0.0 (absent, low, moderate, and high), as well as the association with the levels of depression according to the BDI-SF (no depression, mild, moderate, or severe depression). We also used a Pearson χ2 test to study the association between the groups with no PTSD, partial PTSD, or PTSD and the results of module C of the MINI 5.0.0 and the levels of depression according to the BDI-SF. We used a Pearson χ2 test to check the interdependence between the results of module C of the MINI 5.0.0 and the levels of depression according to the BDI-SF.
Spearman’s correlations were used to determine the strength of the association between the BDI-SF score, the PCL-S score, and the number of significant events and the SSI score and likewise between the BDI-SF score and the number of significant events and the PCL-S score.
Finally, to study the association between posttraumatic stress disorder check list specific (PCLS) and covariables (sex, age, SSI, BDI-SF), odds ratios [OR and 95% confidence intervals (CI)] were estimated using logistic regression.

## Results

### Patients’ Primary Diagnosis and Demographic Features

**Figure 1:**
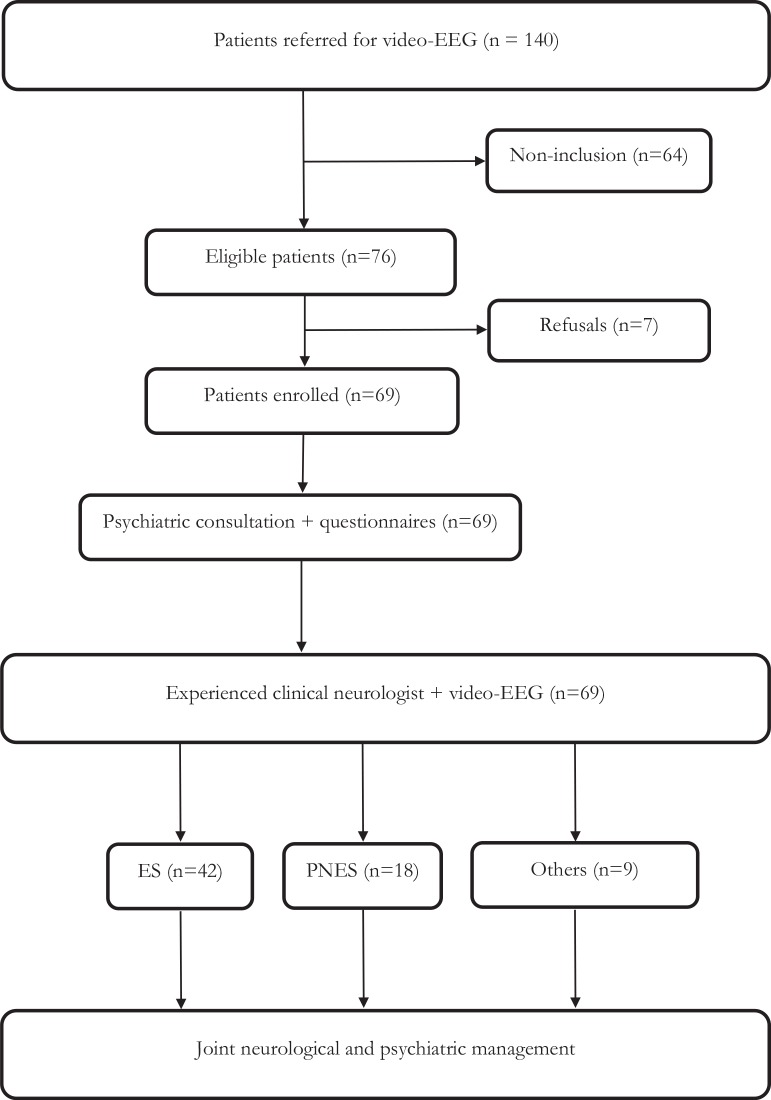
Flowchart.

Of the 76 eligible participants, 7 (9%) refused to participate. Among the 69 participants, 18 (26%) had a diagnosis of PNES and 42 (61%) had a diagnosis of ES. The remaining 9 (13%) included 1 ES/PNES, 1 ES linked to benzodiazepine withdrawal, 1 attention disorder with sleep debt, 1 daytime sleepiness linked to sleep apnea syndrome, 1 cardiac condition, and 4 patients whose final diagnosis remained uncertain despite the global analysis of medical data (interview, EEG, brain MRI, and LMVEEG). The analyses were therefore carried out on 60 participants (**Figure 1**). Among patients with a final diagnosis of PNES, the diagnosis of PNES was already suspected before the LMVEEG in 5 out of 18 patients, and the diagnosis was changed from ES to PNES in the 13 others. Among the 42 patients with a final diagnosis of ES, the diagnosis of possible PNES was suspected in 6 out of 42 patients, and the diagnosis of ES was already clear before the LMVEEG in the 36 others.

In the ES group, the diagnosis was documented in 29 patients, clinically based in 3, probable in 0, and possible in 10 patients. The mean age of this group was 34.1 (SD = 11.4) years and there were 26 females (62%) and 16 males. Eighty-one percent of the patients suffered from drug-refractory epilepsy. The mean duration of epilepsy before LMVEEG was 12.5 (SD = 11.7) years. The patients took an average of 1.93 (SD = 0.7) antiepileptic drugs. Seizure frequency was more than one seizure per day in 3 (7%) patients, one seizure per week in 4 (10%) patients, one seizure per month in 18 (43%) patients, and one seizure per year in 17 (40%) patients. Ninety-five percent were partial epilepsies, with the majority of epileptogenic zones localized in the temporal lobe (67%). A brain lesion could be identified in 40% of the cases. Four (9%) patients took an antidepressant drug on a regular basis.

In the PNES group, the diagnosis was documented in 9 patients, clinically based in 1, probable in 0, and possible in 8 patients. The average age in this group was 30.7 (SD = 13.3) years and there were 13 females (72%) and 5 males. In the PNES group, the mean time lapse between the first seizures and the realization of the LMVEEG was 8.3 (SD = 8.2) years. Eleven (61%) patients regularly took an antiepileptic drug (AED; mean 0.67, SD = 0.6), 2 (11%) of whom were being treated for drug-refractory epilepsy. Seizure frequency was more than one seizure per week in 3 (17%) patients, one seizure per month in 9 (50%) patients, and one seizure per year in 6 (33%) patients. A brain lesion was identified in 3 (17%) patients, and 6 (33%) regularly took an antidepressant drug (**Table 1**). We noted hypermotor symptoms during at least a part of the seizure semiology in 10 out of 18 patients with PNES.

**Table 1:** Demographic and clinical characteristics.

		ES		PNES	Others
**Patients (No.)**		42		18	9
**Sex, no. F/M**		26/16		13/5	9/0
**Mean age (years)**		34.12 (SD = 11.4)		30.7 (SD = 13.3)	38.56 (SD = 11.1)
**Seizure diagnosis (level of certainty)**					
Documented		29		9	
Clinically based		3		1	
Probable		0		0	
Possible		10		8	
**Type of epilepsy: partial or generalized**	Partial (*n* = 40)		Generalized (*n* = 2)		
**Localization of supposed epileptogenic zone (EZ) (% patients)**					
Temporal	27		−		
Frontal	2		−		
Temporo-frontal	5		−		
Other	1		−		
Unknown	5		−		
**Lateralization of the epileptogenic zone (%)**					
Left	19		−		
Right	11		−		
Bilateral	4		−		
Unknown	6		−		
**Lesion**					
Patients with lesion (no.)		17		3	2
Hippocampal sclerosis (no.)		7		1	1
Sequelae (no.)		6		0	1
DNET		2		0	0
Vascular malformations		0		2	0
Anomaly of cortical development		3		0	0
**Seizure frequency**					
>1/day		3 (7%)		0	0
>1/week		4 (10%)		3 (17%)	0
>1/month		18 (43%)		9 (50%)	6 (67%)
>1/year		17 (40%)		6 (33%)	3 (33%)
**Disease duration (y)**		12.5 (SD = 11.7)		8.3 (SD = 8.2)	12.3 (SD = 13.7)
**Pharmacoresistant disease**		34		2	2
**AED (mean no.)**		1.93 (SD = 0.7)		0.67 (SD = 0.6)	1.00 (SD = 1.0)
Lamotrigine		13		2	1
Valproate acid		9		2	2
Oxcarbazepine		8		1	1
Carbamazepine		7		0	0
Benzodiazepines		2		1	1
Levetiracetam		9		3	1
Eslicarbazepine		6		1	0
Gabapentine		0		0	1
Pregabaline		2		1	1
Vigabatrine		1		0	0
Phenitoine		0		0	0
Phenobarbital		0		0	0
Zonegran		7		1	0
Topiramate		5		0	0
Lacosamide		7		1	1
Perampanel		4		1	1
Ethosuximide		1		0	0
**Patients without any AED**		0		6	3
**Patients with antidepressant drug (no.)**		4		6	0

ES, epileptic seizure; PNES, psychogenic nonepileptic seizure, AED, antiepileptic drug.

### Psychiatric Profiles of Epileptic Seizure and Psychogenic Nonepileptic Seizure Groups

The SSI score showed an absence of suicide ideation in 47 (78%) patients, suicide ideation (ranging from 1 to 5) in 8 (13%) patients, and clinically significant suicidal ideation (≥6) in 5 (8%) patients. Suicide risk was rated as absent in 48 (80%) patients, low and moderate in 7 (12%) patients, and high in 5 (8%) patients. The BDI-SF showed an absence of depression in 21 (35%) patients, mild to moderate depression in 36 (60%) patients, and severe depression in 3 (5%) patients. A comparison of these scales showed that suicide ideation scores were positively correlated with symptoms of depression [*r*(60) = 0.47, *p* = 0.001] and the higher the suicide risk, the greater the severity of symptoms of depression [*X* ^2^(9, *N* = 60) = 39.9, *p* < .01]. The same patients (three ES and two PNES) had clinically significant suicidal ideation (≥6) and a high suicide risk (**Table 2**). However, the severity of suicidal ideation and the suicide risk did not differ between the ES and PNES groups. The suicide risk did not differ between patients according to their antiepileptic treatment (containing 0 vs. 1 vs. ≥2 AEDs; **Table 3**). Many patients in both groups had symptoms of depression (64% in ES and 67% in PNES). In the ES group, the BDI-SF showed mild depression in 14 (33%) patients, moderate in 12 (29%) patients, and severe in 1 (2%) patient. In the PNES group, the BDI-SF showed mild depression in 7 (39%) patients, moderate in 3 (17%) patients, and severe in 2 (11%) patients. We observed no difference in the severity of depressive symptoms between the ES and PNES groups: the distribution among the four stages of severity was similar, with no statistical difference in the median severity score (median: 6 vs. 5).

**Table 2:** Severity thresholds with Beck Suicidal Ideation Scale, with MINI 5.0.0, and with Beck Depression Inventory–Short Form in patients with ES and patients with PNES.

	ES (*n* = 42)	PNES (*n* = 18)	Fisher’s exact test
Beck Suicidal Ideation Scale			
0	33 (79%)	14 (78%)	*p* = 0.9
1–5	6 (14%)	2 (11%)	
≥6	3 (7%)	2 (11%)	
Suicide risk with MINI 5.0.0			
Absent	34 (81%)	14 (78%)	*p* = 0.9
Low	4 (10%)	2 (11%)	
Moderate	1 (2%)	0 (0%)	
High	3 (7%)	2 (11%)	
Severity thresholds with Beck Depression Inventory–Short Form			
None	15 (36%)	6 (33%)	*p* = 0.5
Mild	14 (33%)	7 (39%)	
Moderate	12 (29%)	3 (17%)	
Severe	1 (2%)	2 (11%)	
Posttraumatic Stress Disorder Checklist Specific			
Absence of PTSD	40 (95%)	11 (61%)	*p* = 0.001*
Partial PTSD	2 (5%)	4 (22%)	
Probable PTSD	0	3 (17%)	

*Rejection of the null hypothesis with a risk of α error less than 5%.

MINI, Mini International Neuropsychiatric Interview; PTSD, posttraumatic stress disorder.

**Table 3:** Suicide risk with MINI 5.0.0 in patients with no, one, and two or more antiepileptic drugs.

	Antiepileptic drugs			
Suicide risk with MINI	0	1	≥2	Fisher’s exact test
0	4	19	25	*p* = 0.36
≥1	2	4	6	

Three subjects (5%) displayed probable PTSD (all 3 being PNES cases) and 6 (10%) displayed partial PTSD (2 ES and 4 PNES cases). The PNES group reported more traumatic exposures (median: 3 vs. 5; *p* = 0.008) and more PTSD symptoms (median: 17 vs. 27; *p* = 0.001), with a relatively high power (90%; **Table 4**). The levels of depression were more severe in the PTSD group (probable + partial) [*X* ^2^(6, *N* = 60) = 27.1, *p* < .01].

**Table 4:** Suicidal ideation, symptoms of depression, traumatic exposures, and PTSD symptoms in patients with ES and patients with PNES.

	ES (*n* = 42)	PNES (*n* = 18)	Wilcoxon Mann-Whitney test for independent sample
	Median	Median	
SSI^a^	0 (0–0)	0 (0–0)	*p* = 0.96
BDI-SF^b^	6 (2–8)	5 [3–7.8]	*p* = 0.74
No. events^c^	3 (2–4)	5 [2.2–7]	*p* = 0.008*
PCL-S^d^	17 (17–21)	27 [18.8–41.5]	*p* = 0.001*

a Total score on the Beck Suicidal Ideation Scale.

b Total score on the Short-Form Beck Depression Inventory.

c Number of events using the CAPS Life events checklist.

d Total score on the Posttraumatic Stress Disorder Checklist Specific.

*Rejection of the null hypothesis with a risk of α error less than 5%.

Some PNES patients have significant organic lesions, so we checked that we always got the same results excluding these patients. The PNES group (without lesions) reported more exposure to traumatic events (mean: 3 vs. 5.6; *p* = 0.004) and more intense PTSD symptoms (mean: 19.9 vs. 32.8; *p* = 0.001) than the ES group did.

In **Table 5**, we report unadjusted and adjusted odd ratios resulting from the logistic regression models. The PNES group was associated with a significantly increased likelihood of suffering from PTSD symptoms: 5.15 [1.55–20.67], *p* = 0.011. The multivariate analysis indicates that group variables (PNES vs. ES) were still associated with an increased likelihood of PCLS >17: 5.27 [1.43–23.86], *p* = 0.018. Thus, the risk of presenting PTSD symptoms is five times higher in the PNES population than in the ES population.

**Table 5:** Univariate and multivariate logistic regression models for PCLS >17 versus = 17.

	Univariate		Multivariate	
	OR	*p* Value	OR	*p* Value
PNEs vs. ES	5.15 [1.55–20.67]	*p* = 0.011*	5.27 [1.43–23.86]	*p* = 0.018*
SSI^a^	1.09 [0.95–1.37]	*p* = 0.3	0.97 [0.76–1.3]	*p* = 0.79
BDI-SF^b^	1.13 [1–1.31]	*p* = 0.08	1.19 [0.99–1.45]	*p* = 0.08
Sex M vs. F	1.04 [0.36–3.06]	*p* = 0.9	1.91 [0.56–7.07]	*p* = 0.3
Age	0.97 [0.93–1.01]	*p* = 0.19	0.97 [0.92–1.02]	*p* = 0.2

a Total score on the Beck Suicidal Ideation Scale.

b Total score on the Short-Form Beck Depression Inventory.

*Rejection of the null hypothesis with a risk of α error less than 5%.

Life Events from the CAPS most expressed in the PNES population (vs. ES) are “physical assault,” “other unwanted or uncomfortable sexual experience,” “severe human suffering,” and “sudden, unexpected death of someone close to you.” The results show a significant difference for the event “sudden, unexpected death of someone close to you” (*p* = 0.004; **Table 6**).

**Table 6:** Life events from the CAPS in patients with ES and patients with PNES.

	ES			PNES			Fisher’s exact test
	0	≥1	Range	0	≥1	Range	*p* Value
Natural disaster	35	7	0–1	15	3	0–2	1
Fire or explosion	32	10	0–1	13	5	0–1	0.75
Transportation accident	18	24	0–2	9	9	0–4	0.82
Serious accident at work, home, or during recreational activity	34	8	0–2	15	3	0–2	1
Exposure to toxic substance	42	0	0	17	1	0–1	0.3
Physical assault	32	10	0–2	9	9	0–5	0.09
Assault with a weapon	36	6	0–1	14	4	0–2	0.47
Sexual assault	31	11	0–2	11	7	0–1	0.5
Other unwanted or uncomfortable sexual experience	42	0	0	16	2	0–1	0.086
Combat or exposure to a war-zone	39	3	0–1	16	2	0 – 1	0.63
Captivity	41	1	0–1	17	1	0–1	0.5
Life-threatening illness or injury	31	11	0–2	13	5	0–2	1
Severe human suffering	35	7	0–1	11	7	0–4	0.095
Sudden, violent death	35	7	0–1	15	3	0–2	1
Sudden, unexpected death of someone close to you	34	8	0–2	7	11	0–1	0.004*
Serious injury, harm, or death you caused to someone else	0	0	0	0	0	0	
Any other very stressful event or experience	38	4	0–2	15	3	0–1	0.42

*Rejection of the null hypothesis with a risk of α error less than 5%.

### Psychiatric Profile If No Posttraumatic Stress Disorder, Partial Posttraumatic Stress Disorder, or Probable Posttraumatic Stress Disorder

We compared the groups with no PTSD (*n* = 51), partial PTSD (*n* = 6), and probable PTSD (*n* = 3). The patients with probable PTSD had significantly more suicidal ideation and traumatic exposure (**Table 7**).

**Table 7:** Comparison of the groups: absence of PTSD, partial PTSD, and probable PTSD.

	Absence of PTSD (*n* = 51)		Partial PTSD (*n* = 6)		Probable PTSD (*n* = 3)		Kruskal-Wallis test with independent samples
	Mean	Median	Mean	Median	Mean	Median	
SSI^a^	0.6	0	0.2	0	12.3	18	*p* = 0.004*
BDI-SF^b^	5.1	5	7	6.5	15	19	*p* = 0.3
n events^c^	3.3	3	4.5	4	9.3	10	*p* = 0.009*

a Total score on the Beck Suicidal Ideation Scale.

b Total score on the Short-Form Beck Depression Inventory.

c Number of events reported on the CAPS significant events checklist.

*Rejection of the null hypothesis with a risk of α error less than 5%.

The suicide risk was significantly greater in patients with probable PTSD [*X* ^2^(6, *N* = 60) = 14.7, *p* < 0.01], and PTSD symptoms were positively correlated with symptoms of depression [*r*(60) = 0.31, *p* = 0.015].

## Discussion

Despite the small sample size, our study is the first to provide a simultaneous comparison of suicidal ideation, suicide risk, depression disorders, and PTSD between ES and PNES patients examined by vEEG in order to obtain a better definition of the psychiatric profiles of these two groups of patients.

Our results are consistent with most of the studies, although there are few that specifically focused on suicidal ideation in epileptic patients. Suicide risk is higher in epileptic patients: three- to fivefold more than the general population (21), with a higher prevalence of suicidal ideation in epileptic patients (23, 43). This is consistent with the results in our cohort, which showed that 21% of ES patients had suicidal ideation. In addition, the detection of suicidal ideation is well correlated with the assessment of suicide risk. Our work is also the first to assess suicidal ideation in an adult PNES population. The only other measure of this kind concerns a pediatric population (24). Twenty-two percent of our PNES patients had suicidal ideation and 11% of these presented a high suicidal risk.

One finding is that, contrary to pediatric population data, no significant difference was noticed for suicidal ideation and suicide risk in the ES versus the PNES group. These results are consistent with D’Alessio et al. (25), who did not find any difference between PNES and ES in adult populations. Contrary to these two studies, children with PNES reported more suicidal ideation than did children suffering from ES (24). The nonreproducibility of these results in the adult epileptic population might be due to the greater severity of epilepsy in the ES group (higher frequency and intensity of seizures, longer disease history, and probably more treatments) and, consequently, more frequent psychiatric comorbidities and higher suicidal ideation.

Previous studies have already shown that depression is a frequent comorbidity in epileptic patients, especially in drug-refractory epilepsy (20) and in PNES patients (18). Our data are consistent with these results and demonstrate high rates of depressive symptoms in the ES and PNES groups. In most of the studies that compared PNES groups with ES groups, PNES groups tended to have higher rates of depression (18). We noticed no significant difference between these groups in our cohort. However, risks associated with depression are of sufficient concern for these two populations to be taken into account in clinical practice, regardless of whether patients have ES or PNES. It is imperative to treat a possible depressive syndrome to ensure the clinical improvement of epilepsy or PNES.

We observed more traumatic exposure and PTSD symptoms in the PNES group than in the ES group. The three patients who displayed probable PTSD all suffered from PNES. This is also consistent with previous results (6, 7, 10). Patients suffering from PNES generally reported traumatic events (13, 44) with a high prevalence of PTSD (ranging from 33% to 58%) (6, 12, 45). PNES is probably a specific form of dissociation that involves a conversion-like trigger in its manifestation (46) and for many patients is a potentially unrecognized sequelae of traumatic experiences (44). The historical relationship between trauma and dissociation, on one hand, and between trauma and PNES, on the other hand, is compelling evidence that a similar mechanism might be involved. Moreover, levels of depression were more severe in the PTSD group and symptoms of depression were correlated with symptoms of PTSD. Suffering from PTSD increases the risk of depression threefold (47). The severity of suicidal ideation and the risk of suicide in patients with probable PTSD were higher than in the other subjects.

We confirmed that suicidal ideation, suicide risk, symptoms of depression, and PTSD symptoms are frequent and severe in both groups. Therefore, these symptoms require systematic screening in all ES and PNES patients, which can be easily done during the first consultation or during the follow-up with standard validated questionnaires. The management of psychiatric comorbidities and PNES would be easier if the psychiatrist or psychologist met the patient in the vEEG unit and worked in close collaboration with the neurologist, especially during the diagnosis of PNES (48).

Moreover, all patients had a psychiatric interview. They filled the different psychometric scales during this interview. However, we did not choose to implement a qualitative research method with the recording, transcribing, and analysis of maintenance data during the study. However, during psychiatric interviews, epileptic patients seemed to be more likely to think that there was a causal factor between psychic distress and the frequency of their seizures. Conversely, it seemed that patients for whom there was a prior doubt between PNES and epilepsy were more apprehensive to meet the psychiatrist. Indeed, it seems they initially associate the psychiatrist with a questioning of the legitimacy of their suffering and their illness. These points of view seem to be consistent with data found by Whitehead et al. (49). Thus, it seems that the diagnostic announcement has real therapeutic value for PNES patients, as it allows any inconsistencies that they have been subjected to when they have epilepsy patient status to be explained retrospectively (see drug-resistant epilepsy) (50, 51).

Our study suffers from several limitations. Despite detailed investigations, the diagnosis was characterized as only “probable” or “possible” in 10 out of 42 ES patients and in 8 out of 18 PNES patients. Focusing the study on patients with a PNES or ES diagnosis characterized as “documented” or “clinically based” (= exclusion of diagnoses characterized as “probable” or “possible”) might allow more specific and demonstrative results but would have reduced the studied sample to a greater extent. Because of the small cohort, the lack of statistical power in our study might have prevented us from observing a higher level of suicidal ideation in PNES patients in an adult tertiary care epilepsy center. The use of retrospective data of trauma events is also not ideal to investigate the contribution of trauma and PTSD to PNES symptoms (44). However, we succeeded in recruiting a relatively homogeneous cohort of patients in a tertiary care institution. This study is an initial and necessary step for further studies with larger cohorts to confirm or contradict our results. There are different types of ES and PNES (i.e., TLE–AE, TLE non-AE and non-TLE ES, and the five clinical PNES subtypes proposed by Hubsch et al. Therefore, we can assume that these subtypes could have particular psychiatric profiles (52, 53). Consequently, it would be relevant to define *a priori* subgroups in both ES and PNES populations in future trials in order to identify their respective psychiatric comorbidities more precisely.

In our study, the suicide risk in the ES and PNES groups was greater than in the general population and the PNES patients had more severe psychotraumatic symptoms. Suicidal ideation and suicide risk were equally high in the ES and PNES groups. Patients with PNES had been more exposed to traumatic events and had more intense PTSD symptoms. Nevertheless, exposure to traumatic events was also frequent in patients with ES. Moreover, both groups of patients require vigilance and attention in terms of suicidal risk.

## Ethics Statement

This study was approved by the Toulouse University Hospital Ethics and Research Committee No. 04 615.

## Author Contributions

AG, LV, JC, PB, and AY designed the manuscript. AG and VG performed analyses and made the figures/tables. AG, LV, JC, PB, ST, MD, and AY contributed to manuscript design and writing. AG, LV, JC, PB, ST, MD, and AY assisted with study design and were responsible for data collection.

## Conflict of Interest Statement

The authors declare that the research was conducted in the absence of any commercial or financial relationships that could be construed as a potential conflict of interest.
